# Montane Rattlesnakes in México: Venoms of *Crotalus tancitarensis* and Related Species within the *Crotalus intermedius* Group

**DOI:** 10.3390/toxins15010072

**Published:** 2023-01-13

**Authors:** Emily R. Grabowsky, Anthony J. Saviola, Javier Alvarado-Díaz, Adrian Quijada Mascareñas, Kirk C. Hansen, John R. Yates, Stephen P. Mackessy

**Affiliations:** 1 School of Biological Sciences, University of Northern Colorado, Greeley, CO 80639, USA; 2 Department of Biochemistry and Molecular Genetics, University of Colorado Anschutz Medical Campus, Aurora, CO 80045, USA; 3 Department of Molecular Medicine and Neurobiology, The Scripps Research Institute, La Jolla, CA 92037, USA; 4 INIRENA (Instituto de Investigaciones sobre los Recursos Naturales), Morelia CP 58330, Michoacán, Mexico; 5 Biology Department, Tohono O’odham Community College, Tucson, AZ 85634, USA

**Keywords:** enzymes, evolution, mass spectrometry, phenotype, RP-HPLC, SDS-PAGE, snake venom metalloproteinase, toxin, venom

## Abstract

The *Crotalus intermedius* group is a clade of rattlesnakes consisting of several species adapted to a high elevation habitat, primarily in México. *Crotalus tancitarensis* was previously classified as *C. intermedius*, until individuals occurring on Cerro Tancítaro in Michoacán, México, were reevaluated and classified as a new species (*C. tancitarensis*) based on scale pattern and geographic location. This study aimed to characterize the venom of *C. tancitarensis* and compare the venom profile to those of other species within the *Crotalus intermedius* group using gel electrophoresis, biochemical assays, reverse-phase high performance liquid chromatography, mass spectrometry, and lethal toxicity (LD_50_) assays. Results show that the venom profiles of species within the *Crotalus intermedius* group are similar, but with distinct differences in phospholipase A_2_ (PLA_2_), metalloproteinase PI (SVMP PI), and kallikrein-like serine proteinase (SVSP) activity and relative abundance. Proteomic analysis indicated that the highland forms produce venoms with 50–60 protein isoforms and a composition typical of type I rattlesnake venoms (abundant SVMPs, lack of presynaptic PLA_2_-based neurotoxins), as well as a diversity of typical *Crotalus* venom components such as serine proteinases, PLA_2_s, C-type lectins, and less abundant toxins (LAAOs, CRiSPs, etc.). The overall venom profile of *C. tancitarensis* appears most similar to *C. transversus*, which is consistent with a previous mitochondrial DNA analysis of the *Crotalus intermedius* group. These rattlesnakes of the Mexican highlands represent a radiation of high elevation specialists, and in spite of divergence of species in these Sky Island habitats, venom composition of species analyzed here has remained relatively conserved. The majority of protein family isoforms are conserved in all members of the clade, and as seen in other more broadly distributed rattlesnake species, differences in their venoms are largely due to relative concentrations of specific components.

## 1. Introduction

México is the center of rattlesnake diversification, with at least 43 native species, and many of these species are associated with the Mexican Plateau and other high elevation regions [1]. Venoms of many species, especially larger lowland rattlesnakes, have been studied extensively because of the occurrence of human envenomations and the potential for development of therapeutics from venom toxins, but little is known about many smaller, higher elevation rattlesnake species. Several studies have investigated the phylogeny of these species [2,3], but there is a pressing need for research with a focus on ecological relationships and conservation of many Mexican rattlesnake species. In spite of the numerous detailed studies into the proteomes of medically important species [4,5,6], there are still many species, particularly diminutive species of the Mexican highlands, whose venoms are wholly unknown (but see [7]).

The diversity of species in México can be partially attributed to the apparent rapid radiation of rattlesnakes (*Sistrurus* and *Crotalus* genera) throughout México, as suggested by several phylogenetic and biogeographic analyses [2,8,9]. Based on several studies that used various mitochondrial and nuclear markers for investigating phylogeny, the *Sistrurus* and *Crotalus* clade likely originated in the montane pine-oak forests associated with major mountain ranges and then diversified relatively rapidly [2,10]. This idea is supported by the basal position within rattlesnake phylogenies of high-elevation species such as *C. pricei, C. intermedius*, and *C. transversus* [2,10] that occur in highlands habitats. Many of these montane species are endemic to high elevation, often isolated, biodiversity hotspots [11], which is another reason that additional information about these species is necessary for conservation concerns. In addition, a relatively long evolutionary history and isolation may result in local adaptations that are also reflected in venom phenotypes, e.g., [6].

**Figure 1:**
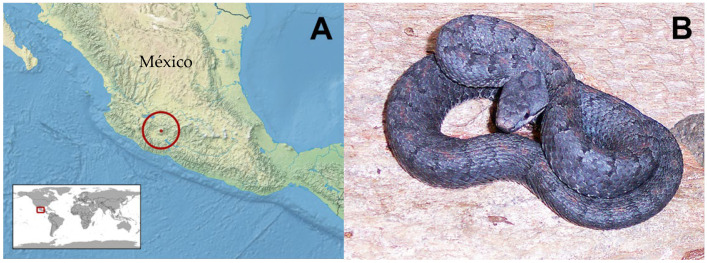
(**A**) Distribution of *C. tancitarensis* (International Union for Conservation of Nature 2007. *Crotalus tancitarensis*. Attribution-Share Alike Creative Commons License). (**B**) Adult female *C. tancitarensis*; photo by SPM.

**Figure 2:**
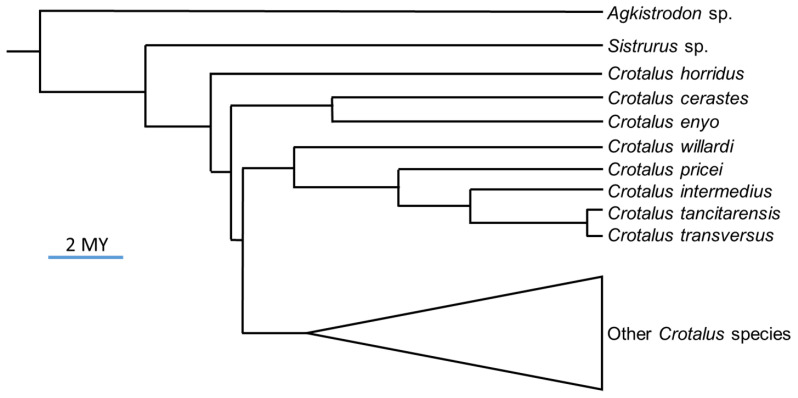
Condensed rattlesnake phylogeny inferred from 6727 bp of combined mitochondrial and nuclear DNA (fossil-calibrated); adapted from Blair and Sánchez-Ramírez [2]. 2 MY, 2 million years.

*Crotalus tancitarensis* is endemic to Cerro Tancítaro in Michoacán, México (Figure 1), and was first officially described by Alvarado-Díaz and Campbell [12]. This species was previously considered to be part of the *C. intermedius* species group, and the first publication to question the identity of this species [13] discussed the geographically isolated nature of this population (nearest population of *C. intermedius* was 300 km away) and several minor morphological features that show similarities to *C. pricei, C. intermedius*, and *C. transversus*. Alvarado-Díaz and Campbell [12] described *C. tancitarensis* using three specimens: one collected in 2002 by Javier Alvarado-Díaz and two collected from Cerro Tancítaro and previously identified as *C. intermedius*. The isolated geographic location and morphological measurements including body length, tail length, fang length, rattle length, and scale counts, were used to distinguish *C. tancitarensis* from *C. intermedius* [12]. More recently, mitochondrial DNA analysis has revealed that *C. tancitarensis* is likely nested with *C. transversus* rather than *C. intermedius* (Figure 2) [2,3], and this relationship may be reflected in venom compositional patterns.

The evolution of venom has allowed for snakes in the families Elapidae and Viperidae, including rattlesnakes, to exploit a chemical means of acquiring and dispatching prey rapidly, as opposed to mechanical methods used by other families such as Pythonidae, Boidae, and many non-rear fanged “colubrid” snakes [14]. These venoms are composed primarily of proteins and peptides and produce a wide array of pathologies [15]. Rattlesnake venoms tend to fall into two categories based on biochemistry, pathology, and toxicity: type I venoms are generally more enzymatic and result in tissue damage caused by high levels of metalloproteinase activity, and type II venoms are highly toxic and cause neurological symptoms due to the presence of presynaptically neurotoxic phospholipase A_2_ toxins [15,16]. Despite this general dichotomy of venom composition and pathology, venoms can be quite variable, and intraspecific variation often exists, likely due to geographic, ontogenetic, and prey variability factors [6,17,18].

Individual species of rattlesnakes often have specific phenotypic characters in the form of dominant venom toxins, and sometimes these can be quite distinct [19]. Often, but not always, more closely related species of rattlesnakes have more similar venoms, and because *C. tancitarensis* is suspected to be closely related to *C. transversus* and *C. pricei*, these venoms may be relatively similar in composition. *Crotalus pricei pricei* venom has been found to have high metalloproteinase activity and moderate toxicity (LD_50_ of 1.25 μg/g) when tested on lab mice [15,20], indicating that it follows the type I venom phenotypic pattern.

This study aimed to analyze venom composition in *C. tancitarensis* and several members of the *Crotalus intermedius* group (including *C. pricei*, *C. transversus*, and *C. intermedius*) using SDS-PAGE, enzyme assays, reverse-phase high performance liquid chromatography, mass spectrometry, and lethal toxicity (LD_50_) assays. By identifying toxins and enzyme activities of *C. tancitarensis* venom and comparing these results to other species within the *Crotalus intermedius* group and with an outgroup (*C. triseriatus*), one can add venom phenotype as an additional set of characteristics to evaluate *C. tancitarensis* relationships to other members of the *C. intermedius* group. To our knowledge, this is the first time that venoms of these taxa (besides *C. pricei*) have been analyzed.

To approach the question of venom characterization of *C. tancitarensis* and where this venom profile falls in the *Crotalus intermedius* group, two hypotheses were tested.

 **H_1_.** *Crotalus tancitarensis venom composition will be most similar to that of C. transversus given its hypothesized position in the Crotalus phylogeny*.

Prediction: Dominant venom phenotypic characteritics, such as metalloproteinase and serine proteinase levels, are predicted to be similar between *C. tancitarensis* and *C. transversus*.

 **H_2_.** *Crotalus tancitarensis venom will be characteristic of type I venom (highly enzymatic; moderate toxicity)*.

Prediction: *Crotalus pricei* produces a venom characteristic of type I venom with moderate to high metalloproteinase activity [15,20]. *Crotalus tancitarensis* is predicted to show similar enzymatic and toxicity patterns.

## 2. Results

### 2.1. SDS-PAGE

**Figure 3:**
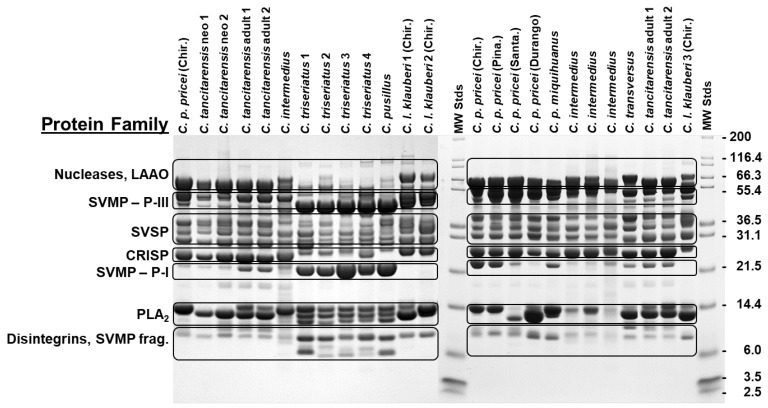
SDS-PAGE gel (12%) of various *Crotalus* species of central México compared to neonate and adult *C. tancitarensis* venoms, and *C. tancitarensis* adult venoms compared to other adult venoms in the *Crotalus intermedius* group. *Crotalus p. pricei* were from various geographic locations: Chiricahua Mountains (Chir.), Pinaleño Mountains (Pina.), Santa Rita Mountains (Santa.), and Durango, México. Approximate molecular mass is displayed to the right and shown in kDa. All proteins were reduced with DTT and visualized using Coomassie Brilliant Blue dye. Typical protein families, as determined by mass and previous experiments with purified toxins, are shown on the left.

Results of SDS-PAGE indicate that *C. tancitarensis* has a venom profile typical of type I venom, with prominent P-III and P-I metalloproteinases (Figure 3). Overall venom profiles appear to be similar between neonate and adult *C. tancitarensis*, with a few key differences. Adults display an obvious PI metalloproteinase band around 21 kDa and a double band around 14 kDa, indicating two compounds in the phospholipase A_2_ (PLA_2_) mass range (Figure 3). Neonate venoms lack both characteristics, with a very faint PI metalloproteinase band and only a single band in the PLA_2_ range (Figure 3). Overall, *C. tancitarensis* electrophoretic patterns are most like that of *C. transversus*, which also displays a PI metalloproteinase band and double PLA_2_ (Figure 3). All venom samples analyzed display a type I venom profile, with outgroups, *C. triseriatus*, and *C. pusillus* also exhibiting toxins in the disintegrin/SVMP fragments range (~6.0–8.0 kDa) (Figure 3).

### 2.2. Enzyme Assays

**Figure 4:**
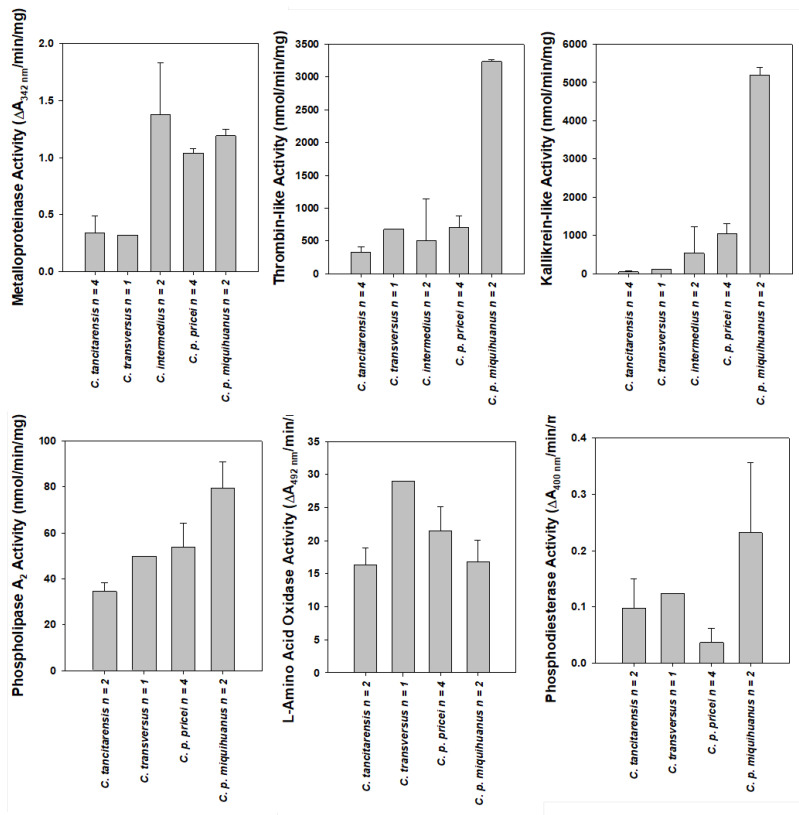
Average enzyme activities of mountain rattlesnake venoms.

Azocasein metalloproteinase enzyme assay results showed a dichotomy between *C. tancitarensis* and *C. transversus* and the rest of the species analyzed. Both *C. tancitarensis* and *C. transversus* had relatively low values of 0.34 and 0.32 A_342 nm_/min/mg, respectively, while the other species analyzed showed activities of 0.95 A_342 nm_/min/mg or higher (Figure 4 and Table 1). *Crotalus tancitarensis* metalloproteinase activity was significantly lower than *C. pricei, C. intermedius*, and *C. p. miquihuanus* (*p* < 0.05).

Enzyme assay results revealed several different enzyme activities between *C. tancitarensis* and closely related species. *Crotalus tancitarensis* had noticeably lower thrombin-like and kallikrein-like serine proteinase activities (SVSP) than other species examined (Table 1 and Figure 4) but was only significantly different from *C. p. pricei* (*p* < 0.05). Specifically, kallikrein-like SVSP activity of *C. tancitarensis* venom was quite low, with a value of 47 nmol/min/mg. *Crotalus p. miquihuanus* crude venom showed exceptionally high activity, with a value of 5201 nmol/min/mg, and was statistically different from *C. tancitarensis, C. p. pricei*, and *C. intermedius* (*p* < 0.01). The second lowest kallikrein-like SVSP value was *C. transversus* crude venom with a value of 112 nmol/min/mg.

Thrombin-like SVSP activities exhibited a similar trend, with *C. tancitarensis* venom showing the lowest value at 328 nmol/min/mg and *C. p. miquihuanus* with the highest value at 3236 nmol/min/mg (Table 1 and Figure 4). *Crotalus intermedius* had the second lowest thrombin-like SVSP activity with a value of 509 nmol/min/mg. *Crotalus p. miquihuanus* was significantly different from *C. tancitarensis*, *C. p. pricei*, and *C. intermedius* (*p* > 0.01).

Phospholipase A_2_ assay results indicated that *C. tancitarensis* had noticeably lower enzymatic PLA_2_ activity (34.6 nmol/min/mg) compared to other species within the *Crotalus intermedius* group but was only statistically significantly different from *C. p. miquihuanus* (*p* < 0.01) (Figure 4 and Table 2). However, the outgroup, *C. triseriatus*, had similar activity at 34.8 nmol/min/mg and was also significantly different from *C. p. miquihuanus* (*p* < 0.01). *Crotalus p. miquihuanus* had the highest PLA_2_ activity with a value of 79.6 nmol/min/mg. The PLA_2_ assay was not completed for neonate *C. tancitarensis* due to insufficient amounts of venom.

Enzyme activity for L-amino acid oxidase (LAAO) indicated that *C. tancitarensis* has moderate enzyme activity of 16.3 A_492 nm_/min/mg compared to other species in the *Crotalus intermedius* group (Figure 4 and Table 2). *Crotalus transversus* exhibited the highest activity with 29.0 A_492 nm_/min/mg. The LAAO assay was not completed for *C. intermedius* or neonate *C. tancitarensis* due to insufficient amounts of venom. There were no significant differences in LAAO activities among the species analyzed.

*Crotalus tancitarensis* phosphodiesterase (PDE) activity assays yielded similar results to those of *C. transversus*. Both species had relatively low PDE activity (0.098 and 0.124 A_400 nm_/min/mg, respectively), though not as low as *C. p. pricei* (0.037) (Figure 4 and Table 2). *Crotalus p. miquihuanus* had the highest activity at 0.232 A_400 nm_/min/mg and was significantly different from *C. p. pricei* (*p* < 0.05), though this is still relatively low compared to PDE specific activity of other *Crotalus* venoms [15]. The PDE assay was not completed for *C. intermedius* or neonate *C. tancitarensis* due to insufficient amounts of venom.

### 2.3. Reverse-Phase High Performance Liquid Chromatography (RP-HPLC)

**Figure 5:**
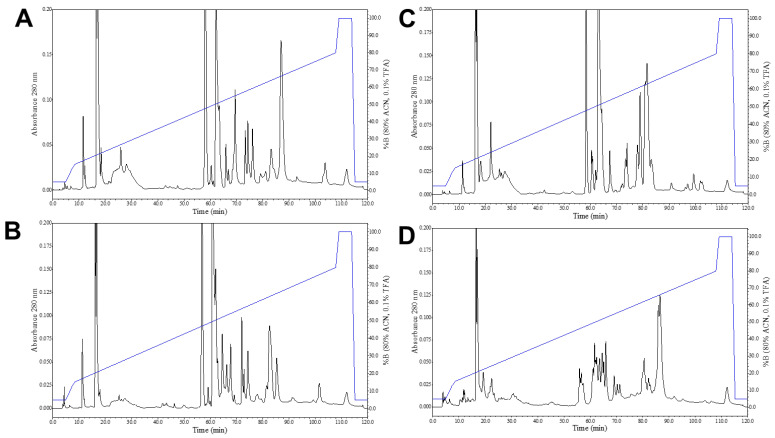
Reverse-phase HPLC chromatograms of *C. intermedius* clade species (2.0 mg venom each). (**A**) *Crotalus tancitarensis* (adult) venom. (**B**) *Crotalus transversus* venom. (**C**) *Crotalus price pricei* venom. (**D**) *Crotalus intermedius* venom. Elution gradient is indicated by the blue line.

Results revealed similar overall venom RP-HPLC profiles among the species examined (Figure 5A–D), with *C. intermedius* being the most divergent. All species displayed a prominent peak between minutes 18–20 (peptides) and a clustering of peaks of varying abundance between minutes 55–90 (enzyme toxins). Overall, *C. tancitarensis* appeared to have a profile most like *C. transversus*, based on peak presence and peak height, particularly between minutes 55–90 where most proteins eluted and most variation between individuals’ venoms appeared.

### 2.4. Mass Spectrometry

**Figure 6:**
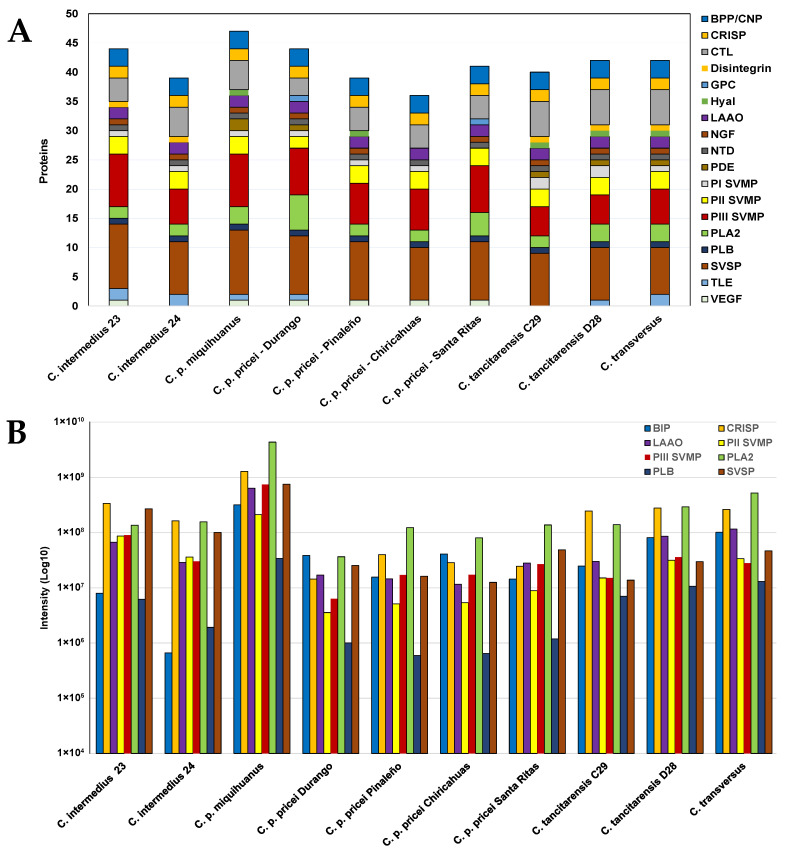
Mass spectrometric analysis of individual venoms. (**A**) Venom protein families and subfamilies identified in each venom by shotgun proteomic analysis. Bars represent total proteins identified, and colors indicate number of proteins in each protein family. (**B**) Summed intensities of peptides per protein family and subfamily for venom proteins. Intensities of each shared peptide in an identified protein were summed based on protein family and subfamily and used for relative quantitative comparison of protein abundances between the different venoms. Abbreviations: bradykinin-potentiating peptides (BPP); cysteine-rich secretory protein (CRISP); C-type lectin (CTL); glutaminyl-peptide cyclotransferase (GPC); hyaluronidase (Hyal); L-amino acid oxidase (LAAO); nerve growth factor (NGF); 5′ nucleotidase (NTD); phosphodiesterase (PDE); PI, PII, PIII snake venom metalloprotease (PI, PII, PIII SVMP); phospholipase A_2_ (PLA_2_); phospholipase B (PLB); (SVSP); thrombin-like serine proteinase (TLE); vascular endothelial growth factor (VEGF).

Mass spectrometric analysis of individual venoms revealed moderate variation in the number of protein families and diversity of isoforms. The number of identified venom proteins ranged from 36 (Chiricahuas *C. p. pricei*) to 47 (*C. p. miquihaunus*) and the number of venom protein (sub)families ranged from 12 (Chiricahuas *C. p. pricei*) to 16 (*C. p. miquihaunus*, Durango *C. p. pricei,* one *C. tancitarensis* individual (D28) and *C. transversus*) (Figure 6A). Overall, 61 unique proteins were identified, 24 of which were shared among all 10 venoms (Supplemental Tables S1 and S2). The protein family with the largest number of proteins was serine proteinases; 11 were identified in *C. p. miquihuanus* venom and an individual *C. intermedius* (23) venom, whereas eight were identified in *C. transversus* venom. Peptides showing sequence similarity to thrombin-like enzymes (TLE) were identified in all venoms except Pinaleño, Chiricahua, and Santa Rita *C. p. pricei* and *C. tancitarensis* (C29) (Figure 6A and Supplemental Table S1). PIII SVMPs were also detected in all venoms and ranged from nine isoforms in one *C. intermedius* (23) individual to five isoforms in the two *C. tancitarensis* venoms. Three PII SVMPs were also identified in each venom except for the Durango *C. p. pricei* venom, which had two, and PI SVMP was detected in all venoms except for the Santa Rita *C. p. pricei*. Both *C. intermedius* venoms and Pinaleño, Chiricahua, and Santa Rita *C. p. pricei* venoms lacked PDEs that were detected in all other venoms. Hyaluronidase was also absent in both *C. intermedius* in addition to the Durango, Chiricahua, and Santa Rita *C. p pricei* venoms, and disintegrin was not detected in all four *C. p. pricei* and *C. p. miquihaunus* venoms. The Chiricahuas *C. p. pricei* venom also lacked a nerve growth factor (NGF) [~Q9DEZ9 *C. d. terrificus*] that was present in all other venoms. PLA_2_s were detected in each venom; both *C. intermedius* venoms, the Pinaleño and Chiricahua *C. p. pricei* venoms, and an individual *C. tancitarensis* (29) venom contained two isoforms, whereas six PLA_2_s were identified in the Durango *C. p. pricei* venom (Figure 6A). A vascular endothelial growth factor (VEGF) [~C0K3N3 *C. atrox*] identified in the venoms of all four *C. p. pricei*, *C. p. miquihaunus* and an individual *C. intermedius* (23) was not detected in any of the other venoms, and a glutaminyl-peptide cyclotransferase (GPC) [~P0CV92 *C. atrox*] was unique to only Durango and Santa Rita *C. p. pricei* venoms. All venoms also contained peptides matching to BPP (3), CRISP (2), LAAO (2), 5′-nucleotidase (NTD) (1), and phospholipase B (PLB) (1) protein families.

Peptide-level analyses identified 778 unique peptides with 78 shared between all ten venoms (Supplemental Table S3). These shared peptides map to BPP [P0CJ34], two CRISPs [~Q7ZT99 and ~F8S0Y4], two LAAOs [~C0HJE7 and ~O93364], two PLA2s [~A0A193CHJ6 and ~Q7ZTA7], PLB [~F8S101], two PII-SVMPs [~C9E1R9 and ~J9Z332], two PIII SVMPs [~F8S108 and ~Q92043], and three SVSPs [~J3S832, ~Q2QA04, and ~Q8QHK3]. For each protein subfamily, intensities of each shared peptide were summed and used as a proxy to compare the abundance of these protein families between the different venoms. All protein (sub)families quantified were most abundant in the *C. p. miquihuanus* venom compared to all other venoms (Figure 6B).

**Figure 7:**
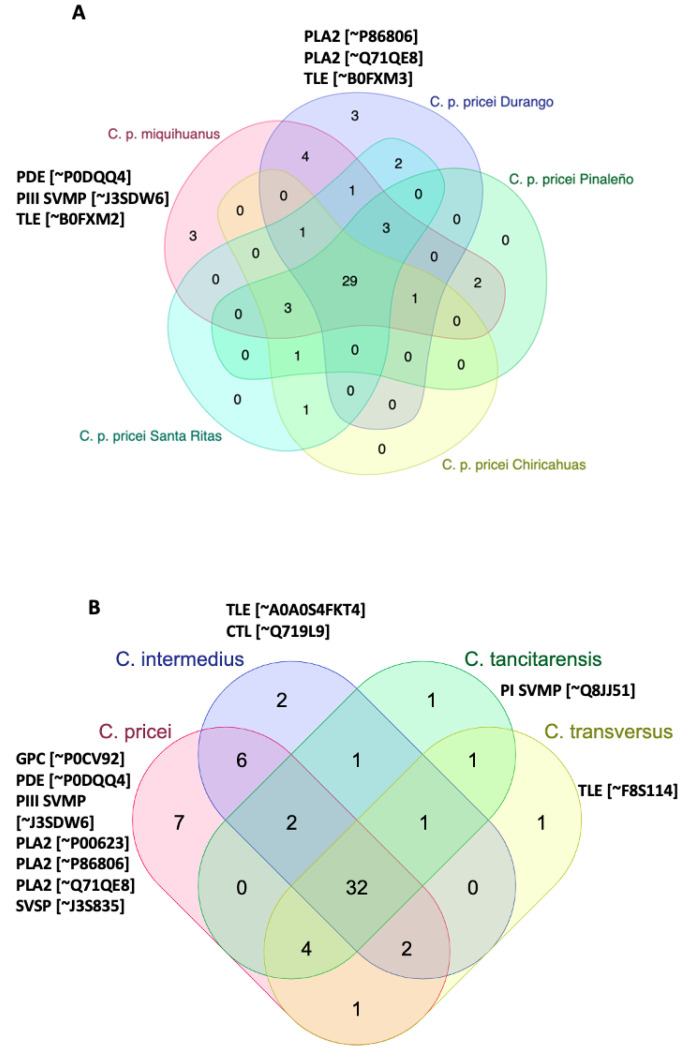
Venn diagrams showing overlapping proteins between the (**A**) *C. pricei* group (the four geographical locations plus *C. p. miquihaunus*) and (**B**) each species. For (**B**), we merged protein identifications for the two individual *C. tancitarensis*, for the two individual *C. intermedius* venoms, and for the five venoms in the *C. pricei* group to permit cross-species comparisons. Toxins unique to each venom are indicated in bold print. Note that in both Venn diagrams, most proteins (29, 32) are shared among all members. Abbreviations: C-type lectin (CTL); glutaminyl-peptide cyclotransferase (GPC); phosphodiesterase (PDE); PI, PIII snake venom metalloprotease (PI, PIII SVMP); phospholipase A_2_ (PLA_2_); serine proteinase (SVSP).

Among the *C. pricei* group (the four geographical locations and *C. p. miquihaunus*), 54 unique proteins were identified (Supplemental Table S1), with 29 being shared between all five venoms (Figure 7A and Supplemental Table S4). A PDE [~P0DQQ4 *C. durissus collilineatus*, ~J3SBP3 *C. adamanteus*], TLE [~B0FXM2 *C. durissus terrificus*], and a PIII SVMP [~J3SDW6 *C. adamanteus*] were unique to *C. p. miquihaunus*, whereas two PLA_2_s [~P86806 *C. durissus cumanensis* and ~Q71QE8 *C. viridis viridis*] and a TLE [~B0FXM3 *C. durissus terrificus*] where unique to the Durango *C. p. pricei* venom (Figure 7A and Supplemental Table S3). All other proteins were shared with at least one other *C. p. pricei* population.

To compare protein profiles across the different species, we merged protein identifications for the two individual *C. tancitarensis*, the two individual *C. intermedius* venoms, and the five venoms in the *C. pricei* group. Thirty-two proteins were shared between the four species (Figure 7B and Supplemental Table S5). Three PLA_2_s [~P86806 *C. durissus cumanensis*, ~Q71QE8 *C. v. viridis*, and ~P00623 C. adamanteus], a PIII SVMP [~J3SDW6 *C. adamanteus*], a GPC [~ P0CV92 *C. atrox*], a PDE [~P0DQQ4 *C. durissus collilineatus*], and an SVSP [~J3S835 *C. adamanteus*] were unique to the *C. pricei* venoms (Figure 7 and Supplemental Table S4). Two proteins that showed sequence similarity to a CTL [~Q719L9 *C. durissus terrificus*] and a TLE [~A0A0S4FKT4 *C. durissus collilineatus*] were detected only in the *C. intermedius* venom, and a PI SVMP [~Q8JJ51 *C. molossus molossus*] and a TLE [~F8S114 *C. adamanteus*] were unique to *C. tancitarensis* and *C. transversus* venoms, respectively (Figure 7B and Supplemental Table S5).

### 2.5. Lethal Toxicity (LD_50_) Assays

**Figure 8:**
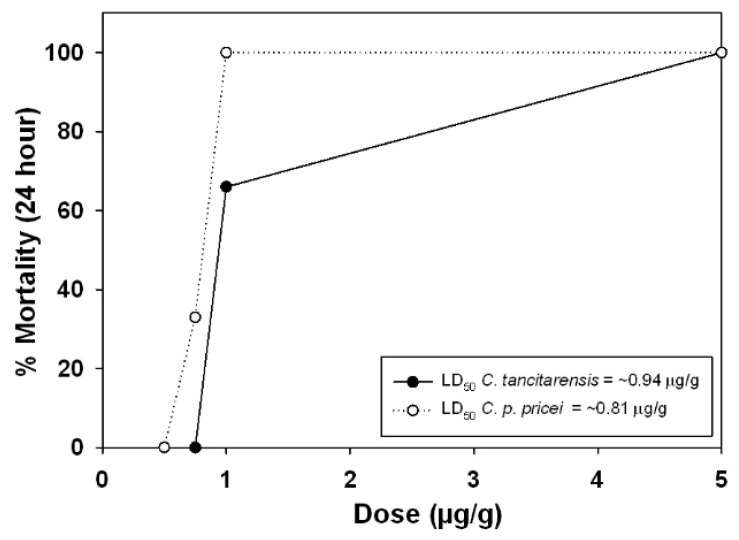
Lethal toxicity (μg/g) of adult *C. tancitarensis* venom and adult *C. p. pricei* venom toward *Hemidactylus frenatus*.

Both *C. pricei* and *C. tancitarensis* displayed high toxicity towards geckos, with LD_50_ values below 1 μg/g (Figure 8). *Crotalus pricei* venom was slightly more toxic towards geckos than *C. tancitarensis*, though the difference is likely not biologically significant. In comparison, the lethal dose of *C. p. pricei* venom (Chiricahuas) when tested on Non-Swiss albino (NSA) mice is 1.25 μg/g [15]. The amount of venom available from *C. tancitarensis* was insufficient to complete LD_50_ assays in NSA mice, but the level of toxicity is likely comparable to *C. p. pricei*, and there is no evidence of β-neurotoxins in the venoms, based on gel electrophoresis, RP-HPLC, or mass spectrometry.

## 3. Discussion

Numerous studies have analyzed species diversification throughout the Mexican highlands [3,11,21,22,23,24,25], and these biodiversity hotspots are home to numerous endemic species, many of which are still being described. During the Last Glacial Maximum (LGM, approximately 23,000–10,000 year BP), the Mexican highland habitat was connected via pine-oak corridors and likely allowed for genetic connectivity between species now located in isolated mountain ranges [21,26,27]. Because of the isolated nature of these communities [28], non-vagile herpetofauna have differentiated substantially since the LGM [2,3,22]. This and earlier periods of differentiation also gave rise to the diversity of rattlesnakes and the isolation of high elevation populations present in the Americas today. However, despite the lack of connectivity between populations of *Crotalus* in the Mexican highlands and Arizona, the venoms of montane species analyzed in this study show relatively little differentiation.

The classification of *Crotalus tancitarensis* as a separate species was initially based on distinct morphological characteristics, scale patterns, and geographic isolation. This isolation has likely existed since the LGM. Along with these defining characteristics, *C. tancitarensis* has a venom profile relatively like species in the *Crotalus intermedius* group (particularly *C. transversus* and *C. p. pricei*), but with some distinct differences. The results of this study support both hypotheses: *C. tancitarensis* venom is similar to other species in the *Crotalus intermedius* group based on SDS-PAGE, RP-HPLC, and mass spectrometry, and this venom displays characteristics of a type I venom; overall, the venom profile of *C. tancitarensis* appears most similar to *C. transversus*. Both species displayed relatively low SVSP (thrombin-like and kallikrein-like) and SVMP (azocasein) activities compared to the other members of the *Crotalus intermedius* group. Conversely, *C. p. pricei* and *C. p. miquihuanus* venom samples displayed high SVSP and SVMP activities compared to other species of rattlesnakes [15,20]. SVSP and SVMP activity results are further supported by mass spectrometry, which demonstrated that PII and PIII SVMPs and SVSPs were most abundant in the *C. p. miquihuanus* venom. Additionally, Saviola et al. [29] found that *C. lepidus* and *C. willardi*, two Sky Island rattlesnakes with similar ecologies to species within the *Crotalus intermedius* group, also have relatively high SVSP and SVMP activity. Low metalloproteinase and low serine proteinase activity is relatively uncommon in species that exhibit no apparent neurotoxic compounds, and these enzymatic toxins are responsible for the tissue degradation and disruption of hemostasis that aid in prey acquisition in species with type I venom [17]. Both enzymatic PLA_2_ activity and PLA_2_ peak intensity were the highest in *C. p. miquihuanus* venom compared to other venoms; however, in general, venom PLA_2_ activity levels are consistent with what has been previously seen with other small, high-elevation rattlesnakes [29].

While overall enzymatic activity in this study is based on combined averages of adult and neonate *C. tancitarensis* venom, ontogenetic shifts in venom composition are common and should be taken into consideration when evaluating venom compositional trends [16,30,31,32]. Neonate *C. tancitarensis* venoms have lower metalloproteinase activity based on faint PI and PIII bands, and the presence of PI, PI-II, and P-III SVMPs in both adult *C. tancitarensis* venoms was confirmed by mass spectrometry. Unfortunately, there was not sufficient neonate venom material to complete PLA_2_ enzyme assays or mass spectrometry, so ontogenetic shifts in the composition of *C. tancitarensis* venom cannot be determined definitively, but based on electrophoretic data, some shifts in activity levels are expected.

Mass spectrometric analysis revealed a high number of venom proteins shared among all members of the *C. intermedius* clade. *Crotalus tancitarensis* and other species within the *C. intermedius* group have venom phenotypes characteristic of type I venoms, with *C. tancitarensis* most similar to *C. transversus*, consistent with close relationships hypothesized on the basis of mitochondrial DNA [2]. Both species lacked glutaminyl cyclase and VEGF, which were present in the *C. p. pricei* venoms, and both species also contained PDE and hyaluronidase, which were not detected in both *C. intermedius* and several of the *C. p. pricei* venoms. PDEs were also not detected in three (Pinaleño, Chiricahua, and Santa Ritas) of the four *C. p. pricei* venoms by mass spectrometry, and enzyme assays showed very low levels of activity. *Crotalus tancitarensis, C. transversus,* and *C. intermedius* venoms also contained disintegrin, which was not detected in any of the *C. pricei* venoms. All other venoms had relatively conserved venom phenotypes with only slight differences in the number of proteins detected within each family and subfamily. Small sample sizes for each group analyzed make it difficult to state unequivocally that the trends observed are typical for entire populations, but given the highly localized distribution of *C. tancitarensis*, the descriptions presented here are very likely representative for this species.

Lack of an apparent neurotoxin and moderately high SVMP activity indicates a type I venom, but *C. tancitarensis* also displays high toxicity toward lizards. *Hemidactylus frenatus* was used as a model for reptilian prey in this study, although recent evidence illustrates that natural prey (*Sceloporus yarrovi*) exhibits higher resistance toward venoms from high elevation *Crotalus* species than does *Hemidactylus* [20]. *Crotalus pricei* was previously shown to be relatively toxic toward Non-Swiss Webster albino (NSA) lab mice as well (LD_50_ = 1.25 μg/g; [15]). Due to insufficient material, mammalian model LD_50_ assays were not completed for *C. tancitarensis*, but the enzymatic activity and venom profiles suggest *C. tancitarensis* venom follows a pattern of toxicity towards mammals similar to that of *C. pricei*. Conversely, type II venoms show very low SVMP activity levels and high lethal toxicity because of the presence of presynaptic neurotoxic PLA_2_s or similar toxins [15,19,33]. Based on these distinctions, the venom profiles of *C. tancitarensis*, *C. transversus, C. pricei,* and *C. intermedius* should be considered type I venoms.

The similarities of *C. tancitarensis* and *C. transversus* venom characteristics complement results of phylogenetic analyses based on mitochondrial DNA and dispersal-extinction-cladogenesis modeling [3] and on a multilocus data set of nuclear and mitochondrial gene sequences [2]. Based on a mixed-model Bayesian approach, *C. tancitarensis* and *C. transversus* create a monophyletic clade that likely diverged from *C. intermedius* during the Pliocene era, between 5.33 and 3.6 million years BP [3,34]. Many of the major mountain ranges throughout México, including those within the Trans-Volcanic Belt, experienced major climatic and vegetation shifts during the Last Glacial Maximum (LGM) around 25,000 years BP [3,26,35]. During this time, the climate of the now xeric Central Mexican Plateau and other similarly warm, dry regions was much cooler and wetter, allowing for movement of species adapted to montane or pine-oak ecosystems to move between ranges. *Crotalus tancitarensis* and *C. transversus* likely diverged much later than Pliocene divergence from *C. intermedius*, possibly during the LGM when *C. p. pricei* and *C. p. miquihuanus* diverged due to geographic isolation of the Sierra Madre Occidental and Sierra Madre Oriental [3]. This could explain the similarities between venom enzymatic phenotypes expressed in *C. tancitarensis* and *C. transversus*, as opposed to the higher SVMP and SVSP enzymatic activities expressed in the rest of the *Crotalus intermedius* group.

Venoms represent trophic adaptations that facilitate prey handling [32,36,37], leading to the evolution of a diverse set of toxic proteins in a given venom [16,38], some of which are clearly more toxic toward specific types of prey than to others [39,40,41]. Among rattlesnakes, lizard-specific toxins are not known, though mammal-specific effects of myotoxin a have been noted [42]. Because venom composition commonly appears to be linked to dominant prey type consumed [32,38,41], it was anticipated that novel toxins could exist among habitat specialists such as *C. tancitarensis*, *C. transversus*, and perhaps other members of the *C. intermedius* clade; however, what was observed was the conservation of a general pattern of venom composition, type I, common to many species of *Crotalus*, including many species that represent a significant human health risk. *Crotalus tancitarensis* and *C. pricei* venoms are somewhat more toxic toward lizards (*Hemidactylus*) than rodents, but we see no evidence of specialized toxins as observed in some rear-fanged snakes, indicating that in spite of occupying a specialized niche, these montane forms produce venoms that are phenotypically similar to more generalized species.

## 4. Conclusions

Venoms from several species of montane rattlesnakes were analyzed, including species whose venoms have never been investigated. Several of these, including *Crotalus tancitarensis*, *C. transversus*, and *C. pricei*, are high elevation lizard specialists, with venoms that are quite toxic to lizard models, suggesting the potential presence of taxon-specific toxins. However, venom proteomes of these ecologically specialized rattlesnakes are phenotypically conservative, implying that the observed type I venoms in this basal clade may be an ancestral trait relative to the more toxic type II venoms. Collectively, rattlesnake venoms are compositionally conservative, and it has been demonstrated repeatedly that primary differences in their venoms largely result from differential expression of amounts and subtypes of common toxin families (e.g., SVMP, SVSP, PLA_2_, CTL, and several others), rather than from the presence of novel toxin families.

## 5. Materials and Methods

### 5.1. Supplies and Reagents

Protein concentration reagents (Pierce BCA Protein Assay kit) and bovine gamma globulin were obtained from Thermo Scientific (Denver, CO, USA). NuPage gels, molecular mass standards, and buffers for electrophoresis were obtained from Life Technologies, Inc. (Grand Island, NY, USA). All reverse phase-high performance liquid chromatography hardware were purchased from Waters Corporation (Milford, MA, USA), and Jupiter 5 µm C_4_ 300 Å 250 × 4.6 mm reversed phase columns were purchased from Phenomenex, Inc. (Torrance, CA, USA). All other reagents (analytical grade or higher) were purchased from Sigma Biochemical Corp. (St. Louis, MO, USA).

### 5.2. Animals and Venoms

*Crotalus tancitarensis* venoms samples were extracted from snakes collected on Cerro Tancítaro and held at INIRENA (Instituto de Investigaciones sobre los Recursos Naturales) in Morelia, Michoacan, Mexico; these were the same individuals used to characterize the species in 2004 [12]. The female was gravid, and both neonate venom samples analyzed were collected from these offspring. *Crotalus pricei* specimens or venoms were collected in the Chiricahua Mountains, Cochise Co., Arizona, Pinaleño Mountains, Graham Co., Arizona and the Santa Rita Mountains, Pima County, Arizona, in accordance with the scientific collecting license guidelines provided by Arizona Game and Fish Department (scientific collection permit #SP591359; SPM). Live *C. p. pricei* were held in the Animal Research Facility (ARF) at the University of Northern Colorado (UNC) (Greeley, CO, USA) and extracted at regular intervals (at least 2 months between extractions). Venom was mechanically extracted from snakes, lyophilized or air dried over desiccant, and stored in a −20 °C freezer until analyzed. In addition, several samples of *C. p. miquihuanus* and *C. p. pricei* venoms were collected from captive snakes at the Chiricahua Desert Museum (Redeo, NM, USA), and a *C. transversus* venom sample was provided by Dr. R. Bryson. *Crotalus intermedius* and *C. triseriatus* venoms were also collected from captive specimens held at INIRENA in Michoacan. Venoms from *C. triseriatus*, likely more closely related to *C. lepidus* than the *C. intermedius* group [2], a sample of *C. pusillus* venom, and several samples of *C. lepidus klauberi* venom (Chiricahua Mtns., AZ, USA) were included in this study (SDS-PAGE) as outgroups. All methods were approved by the UNC Institutional Animal Care and Use Committee (IACUC; protocols 1302D-SM-16 and 1701D-SM-S-20).

### 5.3. Protein Concentration Determination

Lyophilized and air-dried venom samples were dissolved at an approximate concentration of 4.0 mg/mL in Millipore-filtered water. Thermo Scientific Pierce^®^ BCA Protein Assay kit, with bovine gamma globulin as the standard, was used to determine the protein concentration of the crude venom samples, and these values were used in all further assays.

### 5.4. Protein Polyacrylamide Gel Electrophoresis (SDS-PAGE)

Reduced venom samples (20 µg) were electrophoresed on NuPAGE Novex bis-tris 12% acrylamide mini gels with MES running buffer to provide a “molecular fingerprint” comparison of numerous venom samples; Mark 12 standard (7 μL) was run concurrently. Gels were electrophoresed at 150 volts for approximately 90 min and then stained in 0.1% Coomassie Brilliant Blue R-250 and placed on a gyrating shaker overnight. Excess stain was removed the following day and gels were placed in rapid destain (30% methanol, 7% glacial acetic acid in water) for approximately 2 h; gels were then transferred to 7% acetic acid in water and scanned using an HP Scanjet 4570c. Reverse-phase high performance liquid chromatography (RP-HPLC) and SDS-PAGE were used to identify venom toxin families using modified protocols outlined in [43].

### 5.5. Enzyme Assays

Enzymatic activities of crude venoms were determined based on described methods [19,44]. Assays included metalloproteinase, thrombin-like and kallikrein-like serine proteinases, phospholipase A_2_, phosphodiesterase, and L-amino acid oxidase.

### 5.6. Reverse-Phase High Performance Liquid Chromatography (RP-HPLC)

Crude venom samples were analyzed using RP-HPLC. Two milligrams crude venom was resuspended in 200 μL Millipore-filtered water. Samples were then centrifuged at 9500× *g* for 5 min and filtered through a 0.45 µm syringe tip filter before injection onto a Jupiter 5 µm C_4_ 300 Å 250 × 4.6 mm RP-HPLC column. Fractions were collected at a rate of 1.0 mL/min for 120 min. Venoms were fractionated using a gradient of 0.1% trifluoroacetic acid in Millipore-filtered water (solution A) and 0.1% trifluoroacetic acid in 80% acetonitrile in water (solution B). Proteins were eluted with the following gradient: 0–5 min, 95% A and 5% B; 5–10 min, 5–20% B; 10–105 min, 20% to 80% B; and 105–110 min, 80–100% B. The gradient remained at 100% solution B for 5 minutes before returning to the starting conditions of 95% A and 5% B for the remainder of the run. Eluting proteins and peptides were detected at 220 nm and 280 nm. Fractions corresponding to protein/peptide peaks were collected and placed in a −80 °C freezer overnight and then lyophilized.

### 5.7. Mass Spectrometry Analysis

Lyophilized venoms (50 μg) were subjected to trypsin digestion and analyzed by liquid chromatography-tandem mass spectrometry using an Easy nLC 1000 instrument coupled to a LTQ Orbitrap Velos mass spectrometer (both from Thermo Fisher Scientific), as previously described [45]. Tandem mass spectra were interpreted with MSFragger [46] against the UniProt database containing all ‘*Crotalus*’ protein sequences (downloaded 15 October 2021), plus reverse decoys and contaminants. The precursor-ion mass tolerance was 10 ppm and fragment-ion mass tolerance were set to 0.2 Da. Cysteine carbamidomethylation and methionine oxidation were selected as a fixed and variable modifications, respectively. Maximal missed cleavages allowed was two with trypsin specificity and the protein-level false discovery rate (FDR) was ≤1%. Search results were further processed with the Proteomics Assay COMoarator (PACOM) package ([47]; https://github.com/smdb21/PACOM, accessed on 15 November 2022), which utilizes the Panalyzer algorithm to group similar proteins based on shared peptide evidence. PACOM permits distinction between proteoforms, isoforms, and protein family members [47]. Protein family members impossible to differentiate based on peptides assigned to tandem mass spectra were merged into groups, and for clarity, we refer to both single proteins and protein groups as proteins [48,49]. Intensities assigned to each shared peptide in an identified protein were summed based on protein subfamily and used to provide relative quantification between protein subfamilies in each venom.

### 5.8. Lethal Toxicity (LD_50_) Assays

Venom toxicity methods were adapted from [15]; lizards were tested because both *C. pricei* and *C. tancitarensis* are known to be lizard specialists [12,20]. Geckos used in LD_50_ assays were obtained from Bushmaster Reptile (Boulder, CO, USA). Three adult *Hemidactylus frenatus* (2–3 g body mass) were used at each dose level, and three venom samples for *C. p. pricei* (Chiricahuas) and two venom samples for adult *C. tancitarensis* were separately combined in order to obtain an average lethal toxicity measurement for each species. Lyophilized venom was reconstituted in Millipore-filtered water to a concentration of 1.0 mg/mL. Doses appropriately adjusted to individual gecko mass (in 0.9% saline) were injected intraperitoneally anterior to the right hind leg using a 28 gauge × ½ in. needle and 0.5 mL syringe, and a 24-h time frame was used to determine lethal toxicity.

### 5.9. Statistical Analyses

Enzymatic activities were evaluated for significance using a two-way Analysis of Variance (ANOVA) and a Tukey HSD test to determine differences of means between species. *Crotalus transversus* was excluded from analysis due to inclusion of only one sample in the study. *p*-values < 0.05 were considered statistically significant. Due to violation of certain assumptions of ANOVA tests relating to sample size, statistical results should not be considered completely accurate because of low sample numbers in multiple groups.

## Figures and Tables

**Table 1:** Proteolytic enzyme activities of mountain rattlesnake venoms (x ± SD).

Species	Thr (nmol/min/mg)	Kal (nmol/min/mg)	MPr (ΔA_342 nm_/min/mg)
*C. tancitarensis n* = 4	328 ± 87	47 ± 21	0.34 ± 0.15
*C. intermedius n* = 2	509 ± 630	536 ± 697	1.38 ± 0.45
*C. transversus n* = 1	681	112	0.32
*C. p. miquihuanus n* = 2	3236 ± 29	5201 ± 196	1.19 ± 0.06
*C. p. pricei n* = 4	699 ± 182	1055 ± 261	1.04 ± 0.04

Abbreviations: thrombin-like serine proteinase (Thr), kallikrein-like serine proteinase (Kal), and metalloproteinase (MPr).

**Table 2:** Enzyme activities of mountain rattlesnake venoms (x ± SD).

Species	PLA_2_ (nmol/min/mg)	LAAO (ΔA_492nm_/min/mg)	PDE (ΔA_400nm_/min/mg)
*C. tancitarensis n* = 2	34.6 ± 3.8	16.3 ± 2.6	0.098 ± 0.052
*C. intermedius n* = 2	53.8 ± 12.4	--	--
*C. transversus n* = 1	49.8	29.0	0.124
*C. p. miquihuanus n* = 2	79.6 ± 11.4	16.8 ± 3.3	0.232 ± 0.124
*C. p. pricei n* = 4	53.8 ± 10.5	21.5 ± 3.6	0.037 ± 0.025

Abbreviations: phospholipase A_2_ (PLA_2_), L-amino acid oxidase (LAAO), and phosphodiesterase (PDE).

## Data Availability

Data is available by request from the corresponding author.

## Supplementary Materials

The following supporting information can be downloaded at: https://www.mdpi.com/article/10.3390/toxins15010072/s1, Table S1: Full list of peptides identified following shotgun proteomic analysis of all ten venom samples. Table S2. Distribution of identified proteins between all 10 venoms analyzed by shotgun proteomic analysis. Table S3. List of peptides shared between all ten venoms. Table S4. Distribution of identified venom proteins between the *C. pricei* group (the 4 geographical locations and *C. p. miquihaunus*). Table S5. Distribution of identified venom proteins between the four species analyzed by shotgun proteomics.
